# Alteration of cardiac autonomic function in patients with newly diagnosed epilepsy

**DOI:** 10.14814/phy2.12826

**Published:** 2016-06-07

**Authors:** Rajesh K. Goit, Santosh K. Jha, Bhawana N. Pant

**Affiliations:** ^1^ Department of PhysiologyNepalgunj Medical CollegeBankeNepal

**Keywords:** Autonomic nervous system, epilepsy, heart rate variability

## Abstract

The aim of the study was to determine if heart rate variability (HRV) showed any changes in patients with newly diagnosed epilepsy in comparison with controls. Sixty‐five patients with epilepsy (38 males and 27 females), aged 30–50 years, who had never previously received treatment with antiepileptic drugs were eligible for inclusion in this study. Resting electrocardiogram (ECG) at spontaneous respiration was recorded for 5 min in supine position. Time‐domain analysis, frequency‐domain analysis, and Poincare plot of HRV were recorded from ECG. In time‐domain measures, the square root of the mean of the sum of the squares of differences between adjacent RR intervals (RMSSD) and percentage of consecutive RR intervals that differ by more than 50 msec (pNN50) were significantly less in patients with epilepsy. In frequency‐domain measures, high frequency [(HF) msec^2^], HF (nu), and low frequency [LF (msec^2^)] were significantly less in patients with epilepsy while LF (nu) and LF/HF were significantly high in patients with epilepsy. In Poincare plot, standard deviation perpendicular to line of Poincare plot (SD1) and standard deviation along the line of entity in Poincare plot (SD2) were significantly less in patients with epilepsy. Our results suggest that epileptic patients have an impact on the cardiac autonomic function as measured by HRV.

## Introduction

Epilepsy, the tendency to have recurrent unprovoked seizures, is the most common serious neurological disorder. Its prevalence ranges from 0.5% to 1% of the population in developed countries, and is probably higher in developing countries (Banerjee et al. 2009). The mortality rate among individuals with epilepsy is two to three times higher than among the general population (Baker et al. 1997; Annegers et al. 1998). Although autonomic disturbance is a common feature of many seizure types, the signs of autonomic nervous system dysfunction are often overshadowed by the more apparent motor and higher cerebral effects of seizures (Ansakorpi et al. 2000). Partial and generalized seizures often affect autonomic function during seizures as well as during the interictal and postictal periods. Autonomic dysfunction during or after seizures may cause cardiac changes such as cardiac arrhythmias that contribute to sudden unexplained death in epilepsy (Devinsky 2004).

The rhythm of a normal heart is characterized by significant beat‐to‐beat variability. This variability depends on the balance between parasympathetic and sympathetic innervation of the heart. A high variability in heart rate is a sign of healthy heart with well‐functioning autonomic nervous system (Evrengul et al. 2005). This heart rate variability (HRV) has been used as a simple, noninvasive technique to examine autonomic nervous function (Task Force of the European Society of Cardiology and the North American Society of Pacing and Electrophysiology 1996). Regular HRV test provides subclinical early detection of cardiac autonomic neuropathy (Goit et al. 2015). Depressed levels of HRV occur in a number of pathological conditions and predict cardiovascular morbidity and mortality (Kleiger et al. 1987; Huikuri et al. 1998).

Since the autonomic nervous system plays a major role in normal physiological functions and in the pathogenesis of many disorders, measurement of HRV provides an easily applied noninvasive method of assessing cardiac autonomic regulation (Goit et al. 2015). The autonomic nervous system involvement in patients with newly diagnosed epilepsy has rarely been studied and has shown conflicting results. Thus, the aim of this study was to determine if HRV showed any changes in patients with untreated newly diagnosed epilepsy in comparison with normal population.

## Research Design and Methods

### Subjects

A total of 65 patients with epilepsy (38 men and 27 women), aged 30–50 years, who underwent electroencephalography (EEG) and had never previously received treatment with antiepileptic drugs (AED) were recruited from the Department of Physiology at Nepalgunj Medical College, Banke between September 2013 and November 2015. Patients without epilepsy including pseudoseizures, history of cardiovascular disease, diabetes, or any other disease that might affect the autonomic nervous system as well as patients with brain tumors were excluded. Sixty‐five healthy subjects, matched for age and gender with the patients, were studied as the control group. All participants in the study were instructed to avoid alcohol or caffeine‐containing drinks and cigarette smoking during the 12 h preceding the study. The ethical clearance was obtained from the Ethics Committee of the College. Informed consent was obtained from all the participants before commencement.

### Clinical examination

All the subjects were subjected to clinical examination. The individuals with epilepsy were studied during their admission for EEG. At the time of testing, all the subjects were stable in terms of cardiopulmonary function, and showed no withdrawal symptoms. Possible diurnal variation was minimized by performing all tests in the same sequence between 0900 h and 1100 h. Each participant underwent the measurement of his/her weight and height recorded while wearing light indoor clothes but no shoes. Using a tape measure, waist circumference (midway between the lower rib margin and the iliac crest) and hip circumference (the maximal circumference over the buttocks) were measured. Blood pressure was measure using standard protocol.

### Laboratory measurements

The electrocardiogram (ECG) signals for HRV were recorded for five minute using Magic RX (Maestros, India). Subjects had fasted overnight and were kept in the recumbent position throughout the recordings. From ECG, R‐R intervals were measured with a ruler, manually. Then these R‐R intervals were saved as ASCII file. This format was readable by HRV analysis software 1.1 (University of Kuopio, Finland). This HRV analysis software calculates time‐domain results, frequency‐domain results, and nonlinear measures of HRV.

The time‐domain analysis of HRV consisted of the standard deviation of all RR intervals (SDNN); the root mean square of successive differences (RMSSD) of successive normal intervals; and pNN50, which is the proportion of the total RR intervals that have differences in RR intervals >50 msec (Task Force of the European Society of Cardiology and the North American Society of Pacing and Electrophysiology 1996).

The frequency‐domain analysis of HRV consisted of power in high‐frequency range (HF), (0.15–0.40 Hz); power in low‐frequency range (LF), (0.04–0.15 Hz); and power in very low‐frequency range (VLF), (below 0.04 Hz) (Task Force of the European Society of Cardiology and the North American Society of Pacing and Electrophysiology 1996).

It has been speculated that analysis of HRV based on the methods of nonlinear dynamics might elicit valuable information for the physiological interpretation of HRV. One nonlinear method is Poincare plot. The Poincare plot is a scatterplot of the current R‐R interval plotted against the preceding R‐R interval. Using the method described by Brennan (Brennan et al. 2001), these plots were used to extract indexes, such as length (SD2) and width (SD1) of the long and short axes of Poincare plot images.

### Statistical analysis

The clinical characteristics were compared between the groups using Student Independent *T*‐Test and data are presented as mean ± standard deviation (SD). Nonparametric Mann–Whitney *U*‐test was applied for comparisons of the HRV measures and the results are presented as median (interquartile range). A *P* value of <0.05 was considered statistically significant. Data were analyzed with statistical software IBM SPSS Statistics Version 23.

## Results

### Participant characteristics

This study included 65 patients with epilepsy (38 males and 27 females), with age ranging from 30 to 50 years. Forty‐four patients had focal epilepsy and twenty‐one patients had generalized epilepsy. No more than two seizures per patient were included in this study.

The clinical characteristics of the subjects are summarized in Table 1. There were no statistically significant differences between patients and controls in terms of age, height, body mass index, waist hip ratio, and systolic blood pressure. However, diastolic blood pressure, pulse rate, and respiratory rate were significantly higher in patients with epilepsy.

**Table 1:** Clinical characteristics of subjects

Variables	Patients (*n* = 65)	Controls (*n* = 65)
Age (years)	39.23 ± 4.53	40.45 ± 2.76
Sex (Male/Female)	38/27	36/29
Height (cm)	168.45 ± 2.4	167.76 ± 3.4
Body mass index (kg/m^2^)	20.94 ± 1.36	20.95 ± 1.22
Waist hip ratio	0.87 ± 0.22	0.87 ± 0.18
SBP (mmHg)	122.13 ± 6.2	121.87 ± 3.2
DBP (mmHg)	88.13 ± 6	79.33 ± 3.4^a^
Pulse rate (beats per min)	75.06 ± 3.06	70.84 ± 5.2^a^
Respiratory rate (per min)	14.87 ± 4.22	12.22 ± 8.6^a^

SBP, systolic blood pressure; DBP, diastolic blood pressure. Results are expressed as mean ± SD.

a *P *<* *0.05.

### Heart rate variability measures

In time‐domain variables, RMSSD and pNN50 were significantly less in patients with epilepsy than controls. The SDNN was greater in patients with epilepsy, but not statistically significant (Table 2). In frequency‐domain measures, HF (msec^2^), HF (nu), and LF (msec^2^) were significantly less in patients with epilepsy, whereas LF (nu) and LF/HF (%) were significantly higher in patients with epilepsy (Table 2). The variables analyzed in Poincare plot were SD1, SD2, and the ratio of SD2/SD1. SD1 and SD2 were significantly less in patients with epilepsy, but SD2/SD1 was significantly higher in patients with epilepsy (Table 2).

**Table 2:** HRV parameters of subjects

Variables	Patients (*n* = 65)	Controls (*n* = 65)
SDNN (msec)	37 (29–42)	36 (30.75–40)
RMSSD (msec)	25.4 (12.2–36)	35 (24.4–41.8)^a^
pNN50 (%)	3.6 (0.35–14.1)	13.4 (3.85–24)^a^
LF (msec^2^)	66 (24–115)	153 (115–260)^a^
LF (nu)	62.6 (59.3–79.4)	37 (31.3–43.3)^a^
HF (msec^2^)	75 (10–208)	179 (109–264)^a^
HF (nu)	51.4 (30.4–60.95)	62.3 (45.5–69.75)^a^
LF/HF (%)	0.95 (0.64–2.29)	0.61 (0.43–1.2)^a^
SD1 (msec)	16 (9–22.5)	30 (19–38.5)^a^
SD2 (msec)	39 (25–47)	48 (31–66)^a^
SD2/SD1	2.38 (1.49–3.87)	1.68 (1.05–2.47)^a^

SDNN, standard deviation of all RR intervals; RMSSD, the square root of the mean of the sum of the squares of differences between adjacent RR intervals; pNN50, percentage of consecutive RR intervals that differ by more than 50 msec; LF, low frequency; HF, high frequency; nu, normalized unit; SD1, standard deviation perpendicular to line of entity in Poincare plot; SD2, standard deviation along the line of entity in Poincare plot. Results are expressed as median (interquartile range).

a *P *<* *0.05.

## Discussion

This study was designed to find any alternation in cardiac autonomic function in newly diagnosed patients with epilepsy who were not taking AED in the age group of 30–50 years. The findings of this study indicate predictable change in cardiac autonomic activity among patients with epilepsy as measured by HRV.

In time‐domain measures, RMSSD and pNN50 were statistically lower in patients with epilepsy. The RMSSD and pNN50 correlate highly with HF power, reflecting parasympathetic modulation (Cowan 1995). Thus, the time‐domain analysis of HRV showed decreased parasympathetic activity in patients with epilepsy.

In frequency‐domain measures, HF (msec^2^), LF (msec^2^), and HF (nu) were significantly lower in patients with epilepsy, however, LF (nu) and LF/HF (%) were higher in patients with epilepsy. The HF component of HRV is equivalent to spontaneous respiratory sinus arrhythmia and is considered to represent the vagal control of heart rate (Fouad et al. 1984; Saeki et al. 1997; Park and Watanuki 2005). The LF seems to be jointly contributed by both, vagal and sympathetic nerves (Berger et al. 1989). The LF/HF is considered to reflect sympathovagal balance, and acts as an indicator for the sympathetic nervous activity. The LF (nu) is also considered as a marker of sympathetic nervous function. The physiological explanation of VLF alone is not well defined (Task Force of the European Society of Cardiology and the North American Society of Pacing and Electrophysiology 1996). Thus, frequency‐domain analysis of HRV indicates patients with epilepsy have decreased cardiac parasympathetic activity, but increased cardiac sympathetic activity.

In Poincare plot measures, SD1 and SD2 were significantly lower in patients with epilepsy. SD1 mainly describes short‐term variability and SD2 long‐term variability. Analysis of the SD2/SD1 ratio provides information on the relationship between sympathetic and parasympathetic tone. This study showed a higher ratio in patients with epilepsy. A higher SD2/SD1 ratio may reflect an increase in SD2, a decrease in SD1, or both. A decrease in SD1 means a decrease in parasympathetic activity while a decrease in SD2 means an increase in sympathetic activity (Kamen et al. 1996; Tulppo et al. 1996; Toichi et al. 1997). In this study, decrease in SD1 was greater than in SD2, so a higher ratio implies both reduced parasympathetic activity and greater sympathetic activity in patients with epilepsy.

Although the groups were comparable in term of their age, weight, height, body mass index, and waist hip ratio; heart rate is significantly higher in patients with epilepsy. This altered autonomic control of heart rate might be responsible for increased sudden unexplained death in patients with epilepsy.

Several factors are responsible for the sympathovagal imbalance observed in epilepsy during and between seizures. Seizures that arise from or spread to areas in the central autonomic network can mimic stimulation of autonomic afferents or modify autonomic expression (Devinsky 2004). HRV changes during the interictal period might be related to chronic structural changes in autonomic centers, which continuously stimulated or inhibited by repetitive seizures (Wasterlain et al. 1993). Several studies suggest hemispheric lateralization of autonomic cardiovascular control. The right hemisphere may predominantly modulate sympathetic tone, whereas the left hemisphere modulates parasympathetic tone (Wittling et al. 1998).

Devinsky et al. found normal interictal autonomic function in patients with epilepsy (Devinsky et al. 1994). In addition, patients with epilepsy showed a greater variability in blood pressure and heart rate in response to a standard battery of autonomic function tests. Massetani et al. found significantly lower LF and LF/HF ratio in patients with epilepsy which suggests mainly decreased sympathetic tone (Massetani et al. 1997). However, patients with epilepsy showed decreased HF and increased LF in our study, so these differences might be due to the selection of untreated patients in our study. Antiepileptic treatments have a positive effect on sympathetic regulation. Devinsky et al. found that both patients with generalized epilepsy and partial epilepsy develop autonomic dysfunction during the interictal period and seizure control after antiepileptic treatment restores sympathetic autonomic dysfunction in those with partial epilepsy (Devinsky et al. 1994). But, the short‐ and long‐term effects of these treatments on parasympathetic function are poorly understood (Sivakumar et al. 2016). In our study, however, the patient group is fairly homogenous, none of them were receiving antiepileptic treatments, and patient and control groups were age and sex matched.

In conclusion, HRV showed that patients with epilepsy were accompanied by a significant increase in sympathetic control and a significant decrease in parasympathetic control. Hence, our study suggests that cardiac autonomic dysfunction is present in untreated patients with epilepsy.

## Conflict of Interest

None declared.
